# Association of Copy Number Variations in *PTEN* with Autism Spectrum Disorder

**DOI:** 10.3390/genes17101174

**Published:** 2026-09-24

**Authors:** Xu Zheng, Ran Tan, Yuze Wang, Yawen Jiang, Ye Guo, Yi Cheng, Yawen Liu, Shuang Qiu

**Affiliations:** 1 Department of Epidemiology and Biostatistics, School of Public Health, Jilin University, Changchun 130021, China; zhengxu26@mails.jlu.edu.cn (X.Z.); tanran25@mails.jlu.edu.cn (R.T.); wangyuze2021@126.com (Y.W.); jiangyw25@mails.jlu.edu.cn (Y.J.); 2 School of Life Sciences, Jilin University, Changchun 130012, China; yeguo@jlu.edu.cn; 3 Institute of Translational Medicine, The First Hospital of Jilin University, Changchun 130021, China; chengyi@jlu.edu.cn

**Keywords:** autism spectrum disorder, copy number variations, *PTEN*

## Abstract

**Background:** Autism spectrum disorder (ASD) is a complex neurodevelopmental disorder with a strong genetic basis. While copy number variations (CNVs) significantly contribute to ASD risk, the specific role of individual genes within these variants remains unclear. This study investigates the contribution of CNVs in the *PTEN* gene to ASD pathophysiology by integrating population-level genetic analysis with multi-dimensional biological evidence. **Methods:** The biological plausibility of *PTEN* in ASD was first assessed using public multi-omics data, including tissue expression profiles, protein-protein interaction networks, and published cell-type-specific differential-expression data derived from single-nucleus RNA sequencing. Subsequently, a group-matched case-control study was conducted in a Han Chinese population (121 ASD cases and 110 controls) to evaluate the association between *PTEN* CNVs and ASD susceptibility. One-way ANOVA was performed to compare the differences in ASD clinical phenotype scores among groups with different CNV types of *PTEN*. **Results:** Integrative biological analyses revealed that *PTEN* is enriched in ASD-relevant brain regions (e.g., cerebellum) and exhibits cell-type-specific expression in neurons and glia. Furthermore, protein network analysis confirmed its central position within the PI3K/AKT signaling pathway, a critical regulator of neurodevelopment. In the Han Chinese population, no significant association was detected between *PTEN* CNVs and ASD risks or clinical phenotype scores. **Conclusions:** *PTEN* CNVs may not be independently associated with ASD, but may contribute to susceptibility through broader neurodevelopmental networks and molecular pathways.

## 1. Introduction

Autism spectrum disorder (ASD) is an early-onset neurodevelopmental disorder characterized by deficits in social communication and the presence of restricted, repetitive patterns of behavior, with a global prevalence of approximately 1% among children [1]. Family studies consistently demonstrate that ASD exhibits exceptionally high heritability (approximately 80–90%), underscoring the predominant role of genetic factors in its etiology [2]. Current research is limited by the extreme heterogeneity of ASD, the low individual predictive power of common variants, and the pleiotropic nature of rare variants. Although approximately 10–20% of ASD cases can be attributed to identifiable rare genetic variants, and the heritability of ASD is estimated to be as high as 80%, the high heritability has not yet been effectively translated into clearly actionable clinical findings [2,3]. Therefore, expanding our understanding of ASD-associated genetic variants and their functional implications remains a critical priority for advancing biological insights into this disorder.

In recent years, advances in chromosomal microarray analysis and high-throughput sequencing have firmly established CNVs—defined as deletions or duplications of DNA segments larger than 1 kb—as a major source of genetic risk for ASD [4,5,6]. Pathogenic CNVs are estimated to account for approximately 5% to 10% of ASD cases, with a notably higher contribution when the affected genes are critical for neurodevelopment or signaling regulation [7]. Our previous genome-wide CNV analysis in a Northeast Han Chinese population identified multiple rare CNV events and 20 candidate genes associated with ASD risk, including *PTEN* [8]. However, CNV regions typically encompass multiple genes [9]; therefore, the precise contribution of individual genes such as *PTEN* to ASD pathophysiology cannot be determined from CNV data alone. In addition, common variants in other neurodevelopmental genes, such as *RELN*, have been associated with ASD in case-control studies, supporting the relevance of single-gene investigations in ASD [10].

Nevertheless, a substantial body of independent genetic and functional evidence has robustly implicated *PTEN* in ASD [11]. The *PTEN* gene (located at 10q23.3) encodes a tumor suppressor with dual lipid and protein phosphatase activities [12]. It negatively regulates the PI3K/AKT signaling pathway via dephosphorylation of PIP3 to PIP2, a cascade critically involved in neuronal proliferation, synaptic plasticity, and dendritic development [13]. Loss of *PTEN* function results in hyperactivation of this pathway, leading to excessive dendritic growth, aberrant synaptic protein synthesis, and excitatory/inhibitory synaptic imbalance—cellular phenotypes that closely recapitulate the neuropathological hallmarks of ASD [14,15]. Moreover, germline *PTEN* pathogenic sequence variants are detectable in approximately 0.2–1% of ASD patients, and this proportion reaches up to 17% in those with macrocephaly [16]. *PTEN* pathogenic variants are typically sequence-level changes, such as missense, nonsense, frameshift, or splice-site mutations. They directly disrupt *PTEN* protein function and are well established in ASD. Notably, in patients with germline *PTEN* pathogenic variants, the presence of pathogenic or likely pathogenic CNVs was significantly more common among those with ASD/developmental delay, suggesting that CNVs may act as genomic modifiers. [17]. *PTEN* CNVs are larger genomic deletions or duplications that may alter *PTEN* dosage but often span multiple genes. Their functional consequences are more complex and may depend on CNV size, breakpoints, and co-deleted or co-duplicated genes. In this study, we therefore focused specifically on *PTEN* CNVs rather than pathogenic sequence variants.

To further delineate the role of *PTEN CNVs* in ASD, we first integrated publicly available multi-omics data to systematically evaluate its biological plausibility as a candidate gene. Subsequently, we examined the associations between *PTEN* CNVs and ASD susceptibility and clinical phenotypes in a Han Chinese population.

## 2. Materials and Methods

### 2.1. Tissue-Level Expression Analysis of PTEN

To delineate the tissue-level expression landscape of *PTEN*, we obtained gene expression data from a broad spectrum of human tissues via the GTEx Portal (https://gtexportal.org/home/; accessed on 23 March 2026), a comprehensive resource providing bulk RNA-sequencing-based expression profiles across multiple anatomically distinct brain regions and peripheral organs [18]. Specifically, tissue-level *PTEN* expression data were derived from the Genotype-Tissue Expression (GTEx) Project, Analysis V10 (dbGaP accession: phs000424.v10.p2), using gene-level bulk RNA-seq TPM data. Twelve brain regions were analyzed: the amygdala (*n* = 181), anterior cingulate cortex (BA24; *n* = 233), caudate (basal ganglia; *n* = 300), cerebellar hemisphere (*n* = 277), cerebellum (*n* = 266), cortex (*n* = 270), frontal cortex (BA9; *n* = 269), hippocampus (*n* = 255), hypothalamus (*n* = 257), nucleus accumbens (basal ganglia; *n* = 285), putamen (basal ganglia; *n* = 254), and substantia nigra (*n* = 183). In addition, the spinal cord (cervical c-1; *n* = 204) was also included as a central nervous system tissue.

### 2.2. Cell-Type-Specific Expression Analysis of PTEN

To characterize cell-type-specific alterations in *PTEN* expression in ASD, we used the publicly available differential-expression dataset generated by Wamsley et al. (Science, 2024) [19]. Specifically, *PTEN* differential-expression estimates were extracted from Data S1 (“Raw differential gene expression data”), deposited in Dryad (https://doi.org/10.5061/dryad.4b8gthtkr accessed on 23 March 2026). The corresponding cell-type-specific differential-expression data can also be explored through the ASD single-cell browser, the ASD Single-Cell Gene Expression (scGENE) Portal (http://solo.bmap.ucla.edu/asdscgene/ accessed on 23 March 2026). The underlying snRNA-seq study included frontal cortex samples from Brodmann areas 9 and 4/6 from 33 individuals with ASD and 30 control individuals. Following quality control and doublet removal, 591,043 nuclei were analyzed, representing 306,347 cells from ASD subjects and 284,696 cells from control subjects. In the original study, counts from nuclei belonging to the same sample and cell type were aggregated to generate pseudobulk counts and normalized using a variance-stabilizing transformation. Age, postmortem interval, brain region, sequencing batch, mitochondrial RNA percentage, and the number of detected genes were accounted for before differential-expression analysis using limma-voom. In the present study, the published *PTEN* differential-expression estimates were extracted and visualized across major cortical cell types.

### 2.3. PTEN Protein Structure Analysis and Protein–Protein Interactions

Protein-protein interaction data for *PTEN* were obtained from STRING [20]. UsingSelf-Optimized Prediction Method with Alignment (SOPMA), the secondary structure of the *PTEN* protein was predicted [21]. A high-confidence network enriched for ASD-associated genes and PI3K/AKT pathway components was constructed, supplemented by SOPMA protein secondary structure prediction, to define the neurodevelopmental molecular context of *PTEN*.

### 2.4. Association of PTEN CNV with ASD Susceptibility and Clinical Phenotypes

#### 2.4.1. Participants

Children diagnosed with ASD were recruited from Chun guang Hospital (Jilin Province, China) based on DSM-5 diagnostic criteria. Age and sex-matched controls were selected from the same region. A total of 231 participants were included in the study, comprising 121 children with ASD and 110 controls. Written informed consent was obtained from the guardians of all participants prior to study enrollment. The study protocol was approved by the Ethics Committee of the School of Public Health, Jilin University (Approval No:2024-08-08).

#### 2.4.2. DNA Extraction

Peripheral blood samples were collected from each participant, and genomic DNA was extracted using the EE121-01 Blood Genomic DNA Extraction Kit (Beijing Quanshijin Biotechnology Co., Ltd., Beijing, China.). We quantified extracted DNA with an Epoch Take3 microplate reader (BioTek Instruments, Inc., Winooski, VT, USA.), measured absorbance at 260 and 280 nm, assessed DNA purity by the A260/A280 ratio, and selected samples with a ratio of 1.6–1.8 for genotyping. The extracted DNA were stored at −80 °C until further analysis.

#### 2.4.3. *PTEN* CNVs Detection

Specific primers for the *PTEN* gene and the reference gene *GAPDH* were designed for this study, and their sequences are presented in Table 1. Primers targeting exonic regions of *PTEN* and the reference gene (*GAPDH*) were co-amplified. Reactions were conducted on a CFX96 Touch Real-Time PCR System (Bio-Rad Laboratories, Inc., Hercules, CA, USA.) using SYBR Green reagents (Tiangen Biotech, Co., Ltd., Beijing, China) in total volumes of 20 μL which contained 10 μL 2 × SuperReal PreMix Plus, 0.6 μL of each primer, and 2 μL of genomic DNA. The qPCR program consisted of an initial denaturation/enzyme activation step at 95 °C for 15 min, followed by 40 cycles of denaturation at 95 °C for 10 s and annealing/extension at 60 °C for 32 s. The average of values from genomic DNA of reference DNA samples (G1471, G1521, Promega Corporation, Madison, WI, USA.) was considered the normal reference sample for ΔΔCT calculations. Relative fluorescence intensity was then used to calculate copy number ratios. Samples with copy numbers ≤ 1.2 were classified as deletions, while those ≥ 2.8 were considered duplications [22].

#### 2.4.4. Assessment Tools

The Autism Behavior Checklist (ABC) is a widely used screening instrument designed to identify behavioral characteristics associated with ASD. It is commonly employed for adjunctive diagnosis and initial screening. The scale was first developed by Krug and colleagues in 1978 and consists of 57 items that describe atypical behavioral manifestations in children with autism across multiple domains. These items are grouped into five core factors: sensory, relating, body and object use, language, and social and self- help [23].

The Childhood Autism Rating Scale (CARS), developed by Schopler and colleagues in 1980, is a clinical observational instrument used to assist in the diagnosis of ASD and to assess symptom severity. The scale comprises 15 items: relating to people, imitation, emotional response, body use, object use, adaptation to change, visual response, listening response, taste/smell/touch response and use, anxiety reaction, verbal communication, nonverbal communication, activity level, consistency of intellectual response, and general impression. Each item is scored on a 4-point scale ranging from 1 (normal) to 4 (severely abnormal), yielding a total score between 15 and 60 [24].

#### 2.4.5. Statistical Analysis

Continuous variables were described as mean ± SD or *Median* (*P*_25_, *P*_75_), and categorical variables as counts (percentages). Differences in baseline characteristics between groups were compared using the Mann-Whitney U test for continuous variables and the chi-square test for categorical variables. Logistic regression models were used to analyze the association between CNV types and ASD susceptibility, with results reported as odds ratios (*ORs*) and their 95% confidence intervals (*CIs*). One-way ANOVA was performed to compare the differences in ASD clinical phenotype scores among groups with different CNV types of *PTEN*. All statistical analyses were performed using SPSS 24.0 (IBM, Chicago, IL, USA). A two-sided *p* value < 0.05 was considered statistically significant.

## 3. Results

### 3.1. Tissue-Level Expression Analysis of PTEN

Expression of *PTEN* is broadly distributed across diverse human tissues and is consistently observed in multiple brain regions. These include the cerebellum (cerebellar hemisphere and cerebellar tissue proper), cortical areas (anterior cingulate, frontal, and broader cortical regions), and subcortical structures (amygdala, hippocampus, hypothalamus, caudate, nucleus accumbens, putamen, and substantia nigra). Notably, *PTEN* transcript levels are relatively higher within central nervous system tissues than in many non-neural counterparts (Figure 1).

### 3.2. Cell-Type-Specific Expression Analysis of PTEN

Published cell-type-specific differential-expression estimates for *PTEN* were extracted from the Wamsley et al. dataset and summarized across seven major classes of cortical cells, including excitatory neurons (EXT), inhibitory neurons (INT), astrocytes (ASTRO), microglia (MG), oligodendrocyte progenitor cells (OPCS), oligodendrocytes (ODC), and blood-brain barrier (BBB)-associated cells. *PTEN* showed heterogeneous ASD-versus-control differential-expression patterns across these cortical cell populations, with both the direction and magnitude of the changes varying among individual cell-type clusters (Figure 2).

### 3.3. PTEN Protein Structure Analysis and Protein-Protein Interactions

*PTEN* is composed of 403 amino acids arranged in three secondary structural elements; 79 amino acids in the alpha helix (accounting for 19.60%), 74 amino acids in the extended strand (accounting for 18.36%), and 250 amino acids in the random coil (accounting for 62.03%) (Figure 3). The predicted interaction network revealed that *PTEN* interacts with multiple proteins, including PIK3CA, PIK3R1, TP53, DLG1, PTK2, SPOP, PREX2, MAST2, MAGI2, and AKT1 (Figure 4).

### 3.4. Association of PTEN CNV Distribution with ASD Susceptibility and Clinical Phenotypes

#### 3.4.1. Demographic Characteristics

A total of 121 ASD patients and 110 controls were included in this study. The ASD group had a median age of 5.00 (3.00, 6.00) years, with 103 males (85.1%) and 18 females (14.9%). The control group also had a median age of 5.00 (3.00, 6.00) years, with 90 males (81.8%) and 20 females (18.2%). Statistical analysis showed no significant differences between the two groups in age (*Z* = −0.036, *p* = 0.971) or gender distribution (*χ*^2^ = 0.458, *p* = 0.498). These results indicate that the baseline characteristics of the two groups were well matched and comparable, effectively excluding potential confounding effects of age and gender on the subsequent analyses (Table 2).

#### 3.4.2. Association Between CNV Distribution and ASD Susceptibility

The distribution of *PTEN* CNV types in the ASD and control groups is presented in Table 3. In the ASD group, 12.4% had a normal copy number, 84.3% had deletions, and 3.3% had duplications, whereas in the control group, the corresponding proportions were 9.1%, 90.0%, and 0.9%, respectively. No statistically significant associations were observed between *PTEN* CNV types and ASD susceptibility. Compared with the normal CNVs group, deletion was not statistically associated with ASD risks (*OR* = 0.375, 95% *CI*: 0.036–3.865, *p* = 0.410), while duplication was not associated with ASD risks either (*OR* = 0.258, 95% *CI*: 0.028–2.345, *p* = 0.229) (Table 3).

#### 3.4.3. Differences in ASD Clinical Phenotype Scores Among CNVs of *PTEN*

The total ABC score and its five subscales (sensory, relating, body concept, language, and social self-help) were compared among participants with normal, deletion, and duplication CNVs. No statistically significant differences were observed across the three CNVs groups for the total ABC score (*F* = 0.789, *p* = 0.457) or any of the ABC subscale scores (all *p* > 0.05). Similarly, the total CARS score did not differ significantly among the normal, deletion, and duplication CNVs groups (*F* = 2.233, *p* = 0.112) (Table 4). Overall, none of the examined ASD clinical phenotype measures showed statistically significant variation across *PTEN* CNVs categories.

## 4. Discussion

It should be noted that this study did not find a significant association between *PTEN* CNVs and ASD, but this does not negate the biological importance of *PTEN*. To this end, we adopted a multi-evidence integration framework to systematically evaluate the functional link between *PTEN* and ASD.

Tissue-level expression analysis further substantiates the important role of *PTEN* in orchestrating neurodevelopmental processes. Publicly available data from GTEx revealed that *PTEN* exhibits the relatively high expression levels in the cerebellum and its hemispheres, with moderate expression in subcortical nuclei and the hypothalamus -brain regions that correspond to neural substrates repeatedly implicated in mediating the core cognitive, social, and behavioral deficits of ASD [18,25,26]. Specifically, the cerebellum serves as a critical hub for higher-order cognitive functions, including social cognition and emotional regulation. Relevant studies indicate that disruption of cerebellar development during sensitive periods, such as the perinatal and early postnatal stages, can induce persistent alterations in cognitive trajectories, which may underlie later phenotypes associated with ASD [27].

Analysis of the published cell-type-specific differential-expression dataset indicated that *PTEN* showed reduced expression in several astrocytic, oligodendrocyte-lineage, excitatory-neuronal, and inhibitory-neuronal clusters in ASD relative to controls. This suggests that the biological effects of *PTEN* expression abnormalities may not be limited to neuronal signaling. ODC myelination deficits and ASTRO-driven synaptic pruning abnormalities together constitute a microenvironment of disrupted connectivity and impaired plasticity in the ASD brain [28,29]. Consistent with these findings, previous transcriptomic studies of the ASD cortex have shown that the molecular alterations of ASD involve both neuronal and immune-glial modules [30].

Taken together, these findings suggest that the role of *PTEN* in ASD may not arise from drastic changes in a single cell type, but rather from a combined effect on neurons and the surrounding supportive cellular environment, collectively contributing to ASD susceptibility [29]. Cell-type-specific transcriptional alterations may be attenuated in bulk-tissue analyses because signals confined to particular cellular populations can be diluted by cellular heterogeneity. This is likely attributable to the signal dilution effect caused by cellular heterogeneity; the numerically dominant neurons in the prefrontal cortex may mask disease signals originating from glial populations [19,31]. As noted by Bryant et al. (2009), non-responding cells in heterogeneous mixed samples can dilute transcriptional changes in minor cell populations [31].

Furthermore, this study further clarifies that CNVs may contribute to the pathophysiology of ASD not through single-gene effects, but by collectively acting on key signaling pathways [32]. Protein-protein interaction analysis highlighted the pivotal role of *PTEN* in ASD-associated molecular networks. We found that *PTEN* directly interacts with PIK3CA, PIK3R1, and AKT1, while the synaptic scaffold proteins DLG1 and MAGI2 anchor *PTEN* to the postsynaptic membrane via PDZ domains. Other regulatory factors, including PREX2, MAST2, SPOP, and TP53, modulate *PTEN* activity and protein homeostasis. This highly interconnected architecture positions *PTEN* as both a key regulator of the PI3K/AKT/mTOR signaling pathway [33] and a molecular hub for synaptic plasticity [34]-dysfunction of any component may lead to pathway hyperactivation, thereby contributing to ASD pathophysiology [35]. Among these *PTEN*-interacting proteins, PIK3CA, PIK3R1, and AKT1 are canonical components of the PI3K/AKT pathway, and their functional relationship with *PTEN* is well established [36]. TP53, DLG1, and MAGI2 have also been previously linked to *PTEN* through p53 crosstalk or PDZ-domain-mediated synaptic anchoring [37,38,39], and thus represent confirmatory findings. In contrast, the interactions of PTK2, PREX2, MAST2, and SPOP are less well characterized in the context of ASD. Although these interactions have been reported in cancer or general cell signaling studies, their relevance to neurodevelopment and ASD remains largely unexplored. Therefore, these findings should be considered hypothesis-generating and warrant further functional validation in neuronal and glial models.

This study has certain limitations. First, the effect of CNVs is modulated by the genetic background [17], and their functional impact also depends on variant size and the regulatory context [5]. Second, given the relatively limited sample size, this study had limited power to detect small-effect variants and rare *PTEN* CNVs, particularly duplications, and the null findings should therefore be considered preliminary. Future studies incorporating larger sample sizes with transcriptomic and epigenomic data are warranted to further elucidate the functional significance of *PTEN* CNVs and their role in the molecular pathophysiological network of ASD.

## 5. Conclusions

Tissue-level expression, cell-type-specific expression patterns, and the protein–protein interaction network support the role of *PTEN* in ASD. However, in this Han Chinese cohort, *PTEN* CNVs were not significantly associated with ASD susceptibility. *PTEN* CNVs alone may not be an independent causal factor for ASD in this population. The null findings should be considered preliminary given the limited power to detect rare *PTEN* CNVs, particularly duplications. Our findings suggest that *PTEN* likely modulates ASD susceptibility within a broader molecular network.

## Figures and Tables

**Figure 1:**
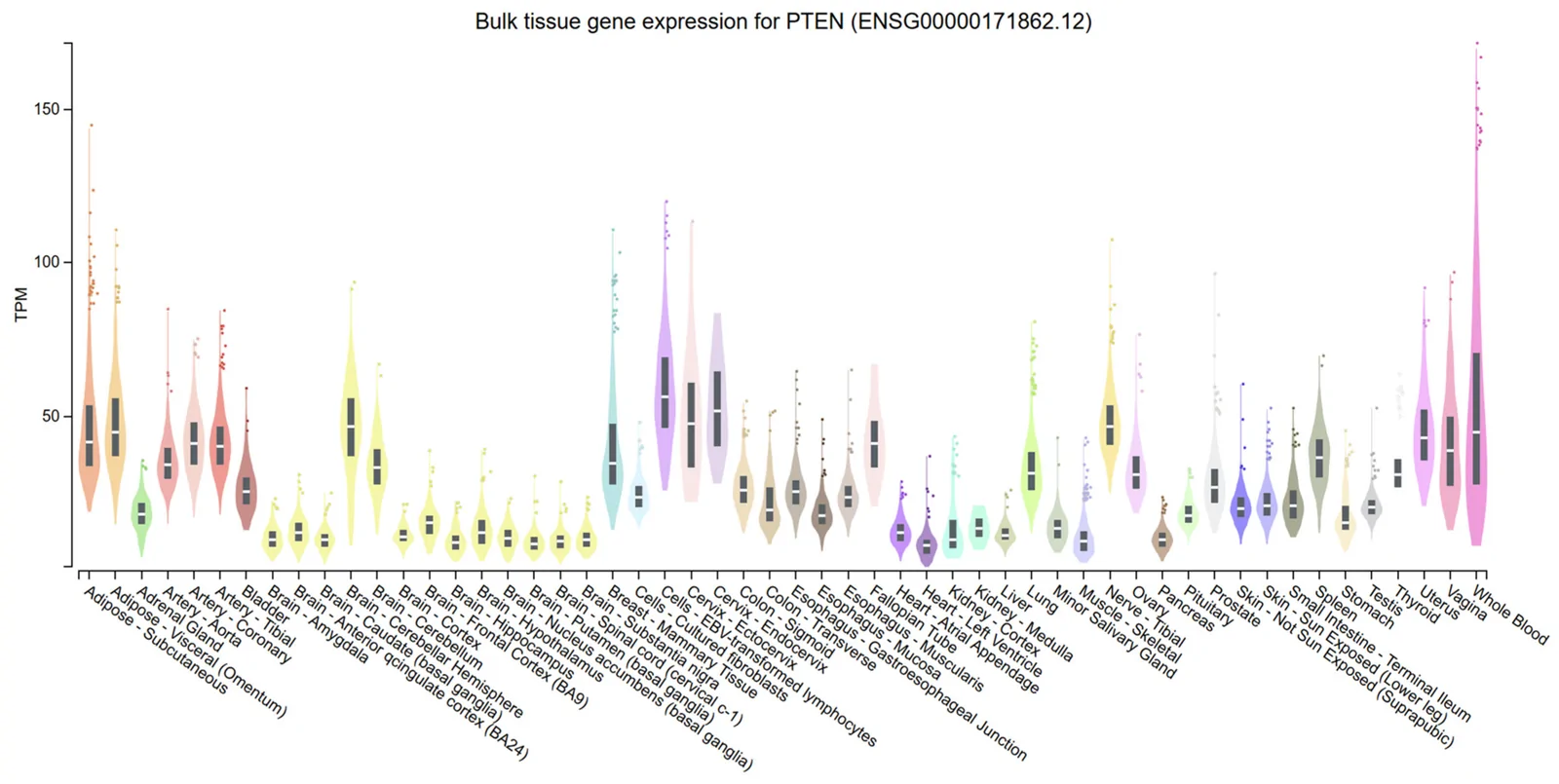
Tissue-level expression profile of *PTEN* across human tissues. *PTEN* tissue-level expression profile were derived from bulk RNA-seq analyses in the GTEx database, with expression levels quantified as normalized TPM (Transcripts Per Million) values and summarized as medians across tissue samples.

**Figure 2:**
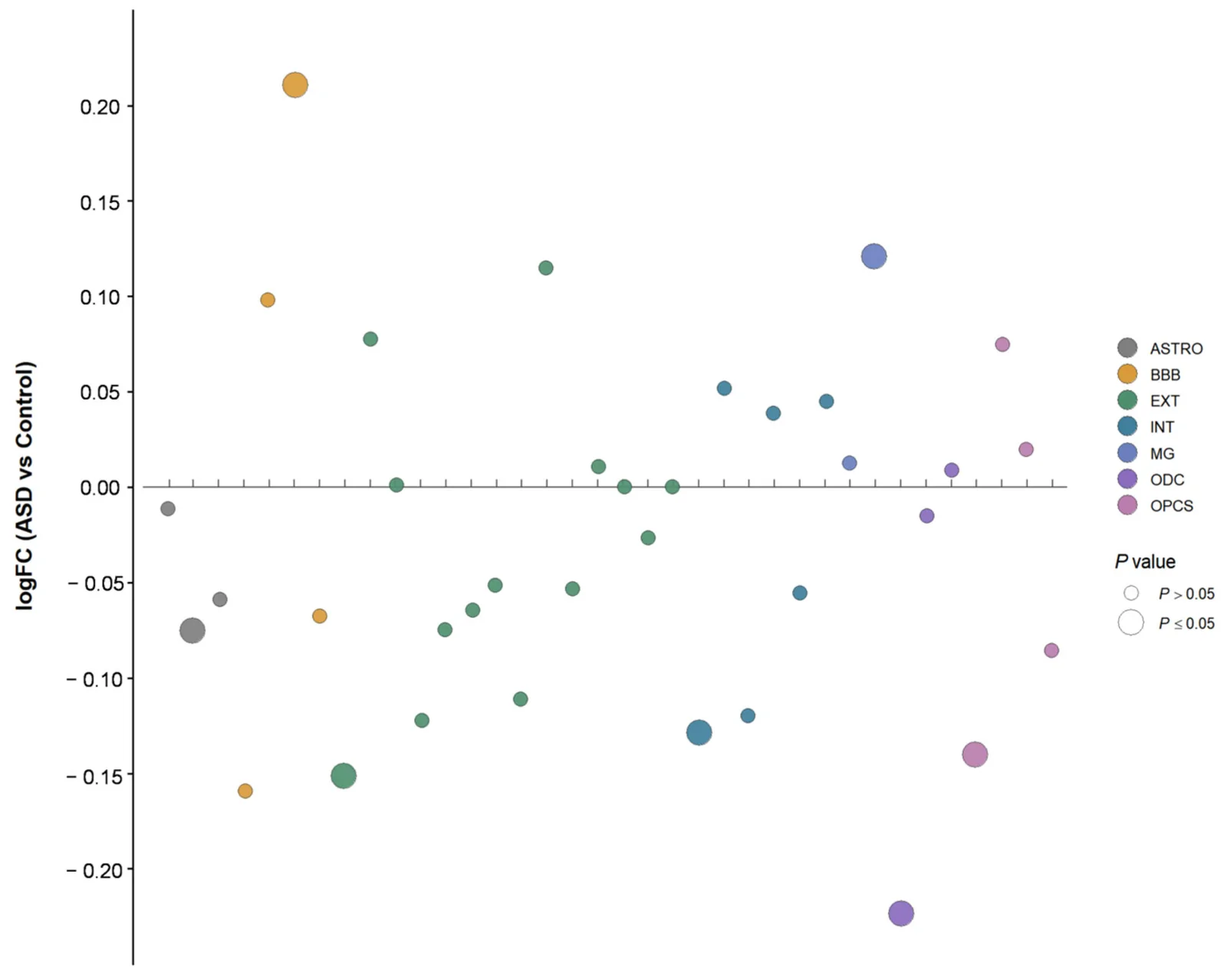
Cell-type-specific differential expression of *PTEN* in the frontal cortex of individuals with ASD versus controls. The *y*-axis represents the log2 fold change, reflecting the logarithmic transformation of the expression ratio (ASD relative to control), where positive values indicate upregulation in the ASD group and negative values indicate downregulation. Each dot represents the log2 fold change in *PTEN* expression between ASD and control samples within a specific cell-type cluster. The size of the dots represents the *p* value, with larger circles indicating *p* ≤ 0.05 and smaller circles indicating *p* > 0.05. ASTRO: astrocytes; BBB: blood-brain barrier; EXT: excitatory neurons; INT: inhibitory neurons; MG: microglia; ODC: oligodendrocytes; OPCS: oligodendrocyte progenitor cells.

**Figure 3:**
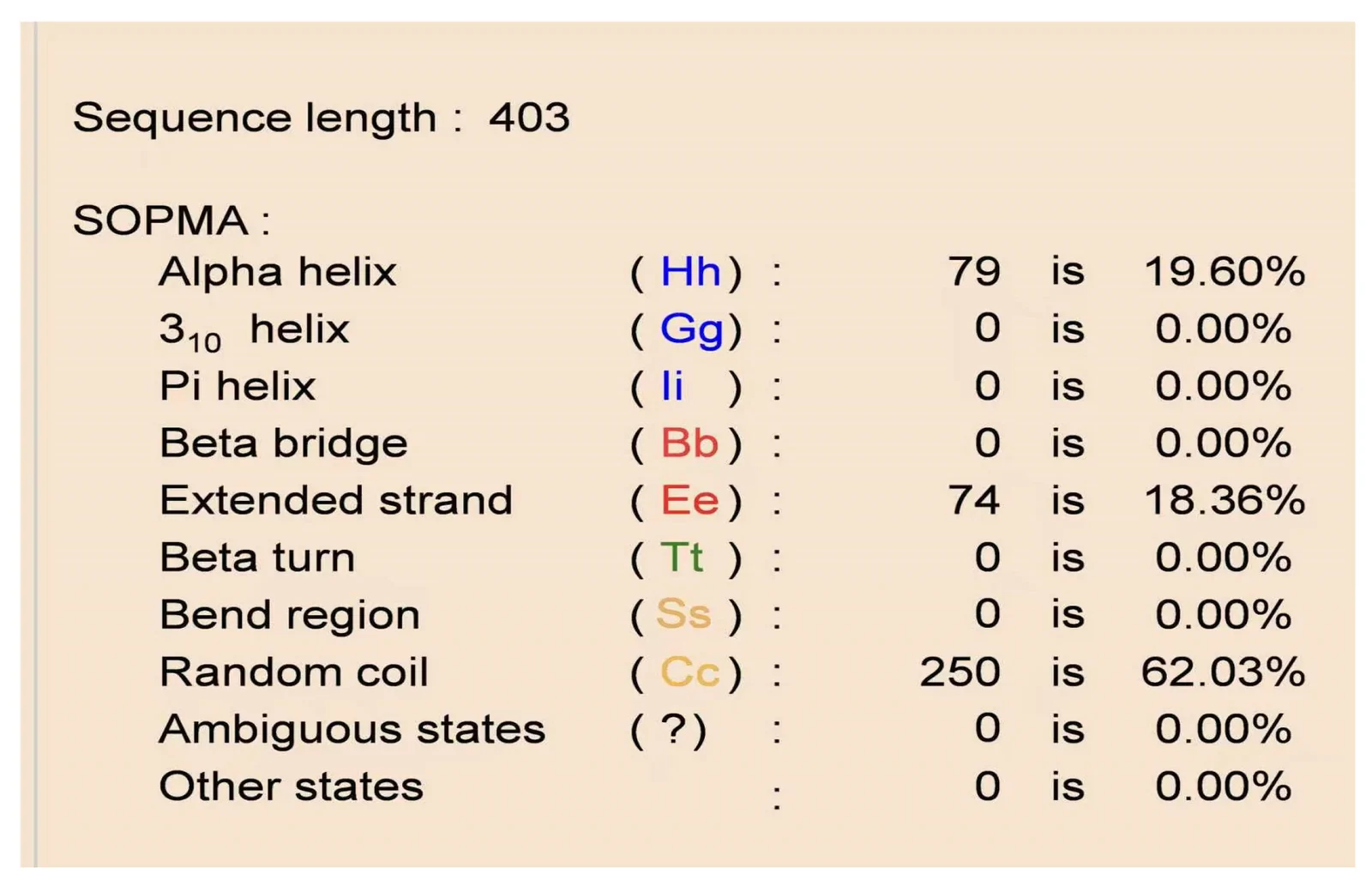
Predicted secondary structure of the *PTEN* protein. Prediction of the secondary structure of *PTEN* was carried out with SOPMA (blue: alpha helix, red: extended, green: beta turn, yellow: random coil). Random coil was the predominant predicted secondary structural element, accounting for 62.03% of the *PTEN* sequence.

**Figure 4:**
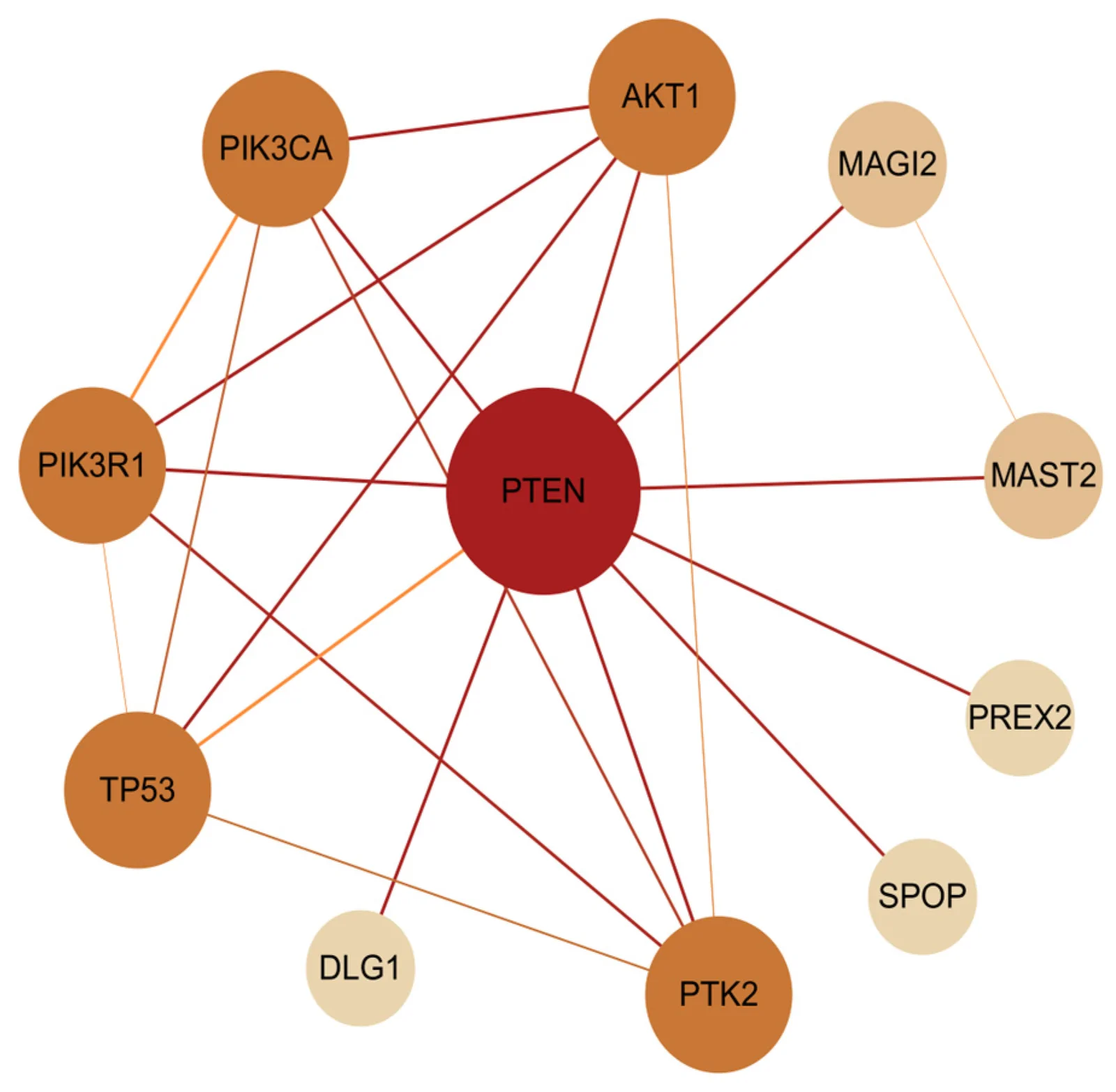
Protein–protein interaction network of *PTEN*. The network illustrates interactions between *PTEN* and key interacting proteins and regulators involved in the PI3K/AKT signaling pathway and related cellular processes, including PIK3CA, PIK3R1, TP53, AKT1, PTK2, MAGI2, MAST2, PREX2, SPOP and DLG1. Nodes represent proteins and edges represent interactions. Node size and color indicate the degree of connectivity, with darker and larger nodes representing higher connectivity. Edge color intensity represents the STRING combined interaction score, with darker edges indicating higher-confidence associations.

**Table 1:** Sequences of DNA primers.

Gene	Primer	Sequences (5′-3′)
*GAPDH*	Forward	CAGGAGGCATTGCTGATGAT
	Reverse	GAAGGCTGGGGCTCATTT
*PTEN*	Forward	TGCGAACGATTGTGATCCGACAG
	Reverse	GCTGCTCAGTGTAGAGGGAAATGC

**Table 2:** Age and gender distribution between ASD and controls [*M* (*P*_25_, *P*_75_), *n* (%)].

Variables	ASD (*n* = 121)	Control (*n* = 110)	*Z*/*χ*^2^	*p*
Age (year)	5.00 (3.00, 6.00)	5.00 (3.00, 6.00)	−0.036	0.971
Gender			0.458	0.498
Male	103 (85.1)	90 (81.8)		
Female	18 (14.9)	20 (18.2)		

**Table 3:** Association between CNVs of *PTEN* and ASD susceptibility [*n* (%)].

CNV Type	ASD (*n* = 121)	Control (*n* = 110)	*OR* (95% *CI*)	*p*
Normal	15 (12.4)	10 (9.1)	Reference	
Deletion	102 (84.3)	99 (90.0)	0.375 (0.036, 3.865)	0.410
Duplication	4 (3.3)	1 (0.9)	0.258 (0.028, 2.345)	0.229

**Table 4:** Differences in ASD Clinical Phenotype Scores Among CNVs of *PTEN* (Mean ± SD).

Items	CNV Type			*F*	*p*
	Normal (*n* = 15)	Deletion (*n* = 102)	Duplication (*n* = 4)		
Items of ABC					
Sensory	13.73 ± 5.35	12.57 ± 5.44	10.75 ± 5.68	0.553	0.577
Relating	18.47 ± 5.78	20.51 ± 5.07	21.25 ± 3.40	1.113	0.332
Body concept	17.07 ± 8.41	20.04 ± 6.52	17.00 ± 4.24	1.579	0.211
Language	9.87 ± 5.68	10.03 ± 4.55	8.25 ± 5.32	0.276	0.759
Social self help	8.87 ± 3.34	9.16 ± 3.91	7.75 ± 4.86	0.279	0.757
ABC Total score	68.00 ± 19.11	72.30 ± 15.74	65.00 ± 19.75	0.789	0.457
CARS Total score	34.20 ± 1.52	35.17 ± 2.45	33.25 ± 2.06	2.233	0.112

## Data Availability

The datasets generated and/or analyzed during the current study are not publicly available due to confidentiality but are available from the corresponding author on reasonable request.

## Abbreviations

ABC	Autism Behavior Checklist
ASD	autism spectrum disorder
CARS	Childhood Autism Rating Scale
CNVs	copy number variations
DNA	deoxyribonucleic acid
mTOR	mammalian target of rapamycin
PIP2	Phosphatidylinositol 4,5-bisphosphate
PIP3	Phosphatidylinositol 3,4,5-trisphosphate
SOPMA	Self-Optimized Prediction Method with Alignment
